# Efficacy and safety of pregabalin for postoperative pain after total hip and knee arthroplasty: a systematic review and meta-analysis

**DOI:** 10.1186/s13018-025-05675-6

**Published:** 2025-03-11

**Authors:** Miguel Ángel Ruiz Ibán, Ángel Oteo-Álvaro, Xoán Miguéns Vázquez, José Luís Ávila, Hermann Ribera, María Pérez-Páramo

**Affiliations:** 1 Shoulder and Elbow Unit, Ramón y Cajal University Hospital, Madrid, Spain; 2 Bone Metabolism Unit. Clínica CEMTRO, Madrid, Spain; 3 Department of Physical Medicine and Rehabilitation, University Clinical Hospital of Santiago de Compostela, Santiago de Compostela, Spain; 4 Upper Limb Unit, Department of Orthopedic Surgery and Traumatology, MAZ Hospital, Zaragoza, Spain; 5 Section of the Pain Unit, Son Espases University Hospital, Palma de Mallorca, Spain; 6 Medical Department, Viatris, Madrid, Spain

**Keywords:** Pregabalin, Neuropathic pain, Total hip arthroplasty, Total knee arthroplasty, Total joint arthroplasty, Postoperative, Meta-analysis

## Abstract

**Introduction:**

The prevalence of osteoarthritis and postoperative neuropathic pain after arthroplasty highlights the necessity for improved pain management. Many patients develop chronic neuropathic pain, necessitating targeted interventions. Research on pregabalin’s effectiveness in pain relief has yielded conflicting findings, necessitating further exploration to determine its therapeutic value. This study sought to assess pregabalin’s efficacy and safety in postoperative pain management, reconcile inconsistent literature, and enhance understanding of its clinical use.

**Methods:**

This study followed the Preferred Reporting Items for Systematic Reviews and Meta-Analyses guidelines. A systematic search was conducted across four major databases to select clinical trials. Statistical analysis was performed using Review Manager 5.4.1, applying fixed- or random-effects models depending on heterogeneity (I^2^). Subgroup analyses were conducted based on the type, timing, and dosage of pregabalin administered.

**Results:**

Pregabalin was associated with significantly reduced pain during movement at 24 h (MD -0.62, 95%CI -1.02 to -0.23), 48 h (MD -0.53, 95%CI -0.90 to -0.15), and 72 h (MD -0.59, 95%CI -1.05 to -0.12) post-surgery. Opioid consumption was also significantly lower at 24 h (SMD − 0.50, 95%CI -0.80 to -0.20), 48 h (SMD − 0.76, 95%CI -1.34 to -0.19), and 72 h (SMD − 1.33, 95%CI -2.16 to -0.49). While there were no significant improvements in the range of motion at 24 and 48 h, pregabalin was associated with significantly enhanced range of motion at 72 h (SMD 1.11, 95%CI 0.12, 2.09). Treatment with pregabalin was associated with a significant decrease in the odds of nausea (OR 0.30, 95%CI 0.09 to 0.99) and vomiting after total knee arthroplasty (TKA) (OR 0.17, 95%CI 0.04 to 0.65). Additionally, pregabalin exposure was associated with increased sedation after TKA (OR 2.27, 95%CI, 1.13 to 4.56) and total hip arthroplasty (THA) (OR 2.54, 95%CI 1.11 to 5.79), as well as blurred vision at 24 h in TKA/THA patients (OR 4.68, 95%CI 1.37 to 15.99; *n* = 95; I2 = 34). There was no significant association with other adverse events. The administration of pregabalin for more than 24 h before surgery was associated with maximal reductions in pain and opioid use at 72 h post-surgery.

**Conclusion:**

Pregabalin was associated with significantly reduced postoperative pain and opioid use following total joint arthroplasty while enhancing mobility on the third day, with acceptable tolerability and safety.

**Supplementary Information:**

The online version contains supplementary material available at 10.1186/s13018-025-05675-6.

## Introduction

The global prevalence of hip osteoarthritis is 9%, increasing to 15% for knee osteoarthritis, and reaching nearly 20% in certain subgroups [1, 2]. The prevalence has been increasing over the years [3]. After Total Knee Arthroplasty (TKA), over half of the patients reported their worst pain during the first two weeks, with many seeking additional help due to insufficient pain management information [4]. Effective preoperative pain control is vital for postoperative outcomes [5]. Chronic neuropathic pain, affecting 10–50% of patients, requires preventive strategies [6]. Multimodal management, focusing on drugs targeting various pain mechanisms and reducing opioid use, is essential [7]. Pregabalin acts by binding to the α2δ-1 subunit of voltage-dependent calcium channels, reducing excitatory neurotransmitter release and decreasing neuronal excitability [8].

Research on the impact of pregabalin on postoperative neuropathic pain management after total joint arthroplasty has yielded mixed results. Some authors have reported the benefits of pregabalin in the short- or long-term, while others found immediate benefits of pregabalin that did not last beyond six weeks or three months, while others found beneficial effects with low doses of pregabalin or directly, and they did not report beneficial effects of pregabalin [9–14].

Pregabalin has demonstrated significant benefits in both orthopedic and non-orthopedic surgeries. In orthopedic procedures such as spinal and upper extremity surgeries, pain is reduced on the VAS scale [15, 16]. Anterior cruciate ligament surgery decreases opioid consumption [17] and improves analgesia in tibial fracture surgeries [18]. In non-orthopedic surgeries, it shortens hospitalization in cardiac surgeries [19], reduces neuropathic pain in breast cancer surgeries [20], and alleviates opioid side effects after hysterectomy [21].

Previous meta-analyses have reported several limitations. Mao et al. found that gabapentinoids reduced opioid consumption, although few studies were included [22]. Dong et al. highlighted the safety of gabapentinoids, particularly their low incidence of nausea and vomiting, suggesting an additional benefit [23]. Hamilton et al. observed no significant differences in neuropathic pain control or range of motion between gabapentin and pregabalin [24]. However, important factors remain underexplored, such as the optimal dosage, drug combinations, and reasons for patient dropout [25]. Li et al. emphasized the need for further research on dosage to improve pregabalin treatment outcomes [26]. Due to the controversy surrounding pregabalin’s efficacy, Clark et al. called for a reevaluation of its role in postoperative pain management [10], noting that many meta-analyses rely on older data from to 2015–2016. Furthermore, inadequately managed postoperative neuropathic pain contributes significantly to global health costs, especially in the context of an aging population and rising disability-related health expenses [27, 28].

The main objective of this study was to comprehensively evaluate both the efficacy and safety of preemptive pregabalin in the management of acute postoperative pain in patients who underwent total hip or knee arthroplasty.

## Methods

### Eligibility criteria

This study had a written protocol with review questions, search strategy, inclusion/exclusion criteria, and risk of bias assessment. The study protocol was conducted with strict adherence to the Preferred Reporting Items for Systematic Reviews and Meta-Analyses (PRISMA) guidelines [29]. Utilizing the PICOS framework to identify articles focused on the management of postoperative pain in patients undergoing total joint arthroplasty (TJA), this study focused on adult patients undergoing total knee arthroplasty (TKA) or total hip arthroplasty (THA). The I—intervention analyzed was the administration of pregabalin compared to either placebo or other conventional pain management medications (C). The primary outcomes assessed were the efficacy and safety of pregabalin, with the S—design limited to randomized clinical trials (RCT). Exclusion criteria were rigorously applied to ensure study integrity, including the elimination of duplicates and non-randomized studies, such as editorials, case reports, series, cohort studies, case-control studies, cross-sectional studies, and protocols. Additionally, studies involving non-adult populations or those with incomplete or missing data were excluded.

### Information sources and search methods for identification of studies

The literature search was conducted across multiple databases, including PubMed, Scopus, and the Cochrane Library, in October 2024, without any restrictions on publication date or language. The search strategy employed involved keywords and phrases such as Pregabalin OR Lyrica, combined with various terms related to joint arthroplasty including “total joint arthroplasty,” TJA, “arthroplasty,” “knee arthroplasty,” “hip arthroplasty,” “hip replacement,” “knee replacement,” “joint replacement,” “TKA,” and “THA” (detailed in Additional file 1). A manual search of the references was conducted to ensure a comprehensive coverage of the literature. No grey literature was included in the search to maintain the scientific rigor of the sources. The initial selection of studies was performed independently by two reviewers to ensure objectivity. Any discrepancies or disagreements between the reviewers were resolved through discussion with a third reviewer, guaranteeing a thorough and unbiased review process.

### Data extraction and data items

Data extraction was performed by two reviewers, and any disagreements were resolved by consulting a third reviewer to ensure consistency and accuracy. The baseline characteristics collected included country, follow-up duration, sample size, age, proportion of female participants, type of total TJA, dosages of pregabalin or control, conflict of interest (COI), and funding sources. The primary efficacy outcome measures were the Visual Analog Scale (VAS) score for pain at rest and during movement, opioid consumption, and range of motion, which included details such as passive flexion. These variables were measured 24, 48, and 72 h postoperatively. Adverse events were also recorded, along with the percentage of patients who discontinued the study, whether due to any cause, or specifically due to adverse events or inadequate pain control.

### Assessment of risk of bias in included studies

The risk of bias in the study was assessed using the Cochrane Collaboration tool (RoB 2) with analyses conducted using Review Manager 5.4.1 software [30]. This assessment included six domains: random sequence generation, allocation concealment, blinding of participants and personnel, blinding of outcome assessments, incomplete outcome data, and selective reporting. Each domain was classified as having low, high, or unclear risk of bias.

### Assessment of results

Statistical analysis was conducted using Review Manager, version 5.4.1. Odds ratios (ORs) with 95% confidence intervals (CIs) were calculated for the dichotomous variables. Continuous variables were analyzed using mean differences (MDs) with 95% confidence intervals (CIs), and standardized mean differences (SMDs) with 95% CIs were used in cases where studies reported outcomes using incompatible units or scales. Heterogeneity among the studies was assessed using the chi-square statistic and I^2^ test, with I^2^ values of 25%, 50%, and 75% indicating low, moderate, and high levels of heterogeneity, respectively. Based on the level of heterogeneity, a fixed-effects model was used when no significant heterogeneity was observed. Conversely, a random-effects model was employed when heterogeneity was detected (I² ≥ 50%). For studies presenting results in graphical form, WebPlotDigitizer software version 4.5 was employed to extract the data. Missing data were handled according to the guidelines set forth in the Cochrane Handbook, ensuring a methodologically sound approach to data integration and interpretation [31].

### Publication bias

Publication bias was assessed using Review Manager version 5.4.1 through the creation of funnel plots, and visual inspection was conducted to evaluate the symmetry of these plots.

### Additional analyses

Subgroup analyses were conducted based on the type of TJA, categorizing studies into those involving TKA, THA, or both TKA/THA. Subgroup analyses were also conducted based on the timing of pregabalin administration (induction, 1–2 h, 8–12 h, and > 24 h).

To evaluate the certainty of the results, the Grading of Recommendations Assessment, Development, and Evaluation (GRADE) system was employed using GRADEpro software. This approach assesses the quality of evidence and strength of recommendations by considering factors like study design, risk of bias, inconsistency, indirectness, imprecision, and publication bias [32].

## Results

### Study selection

**Fig. 1:**
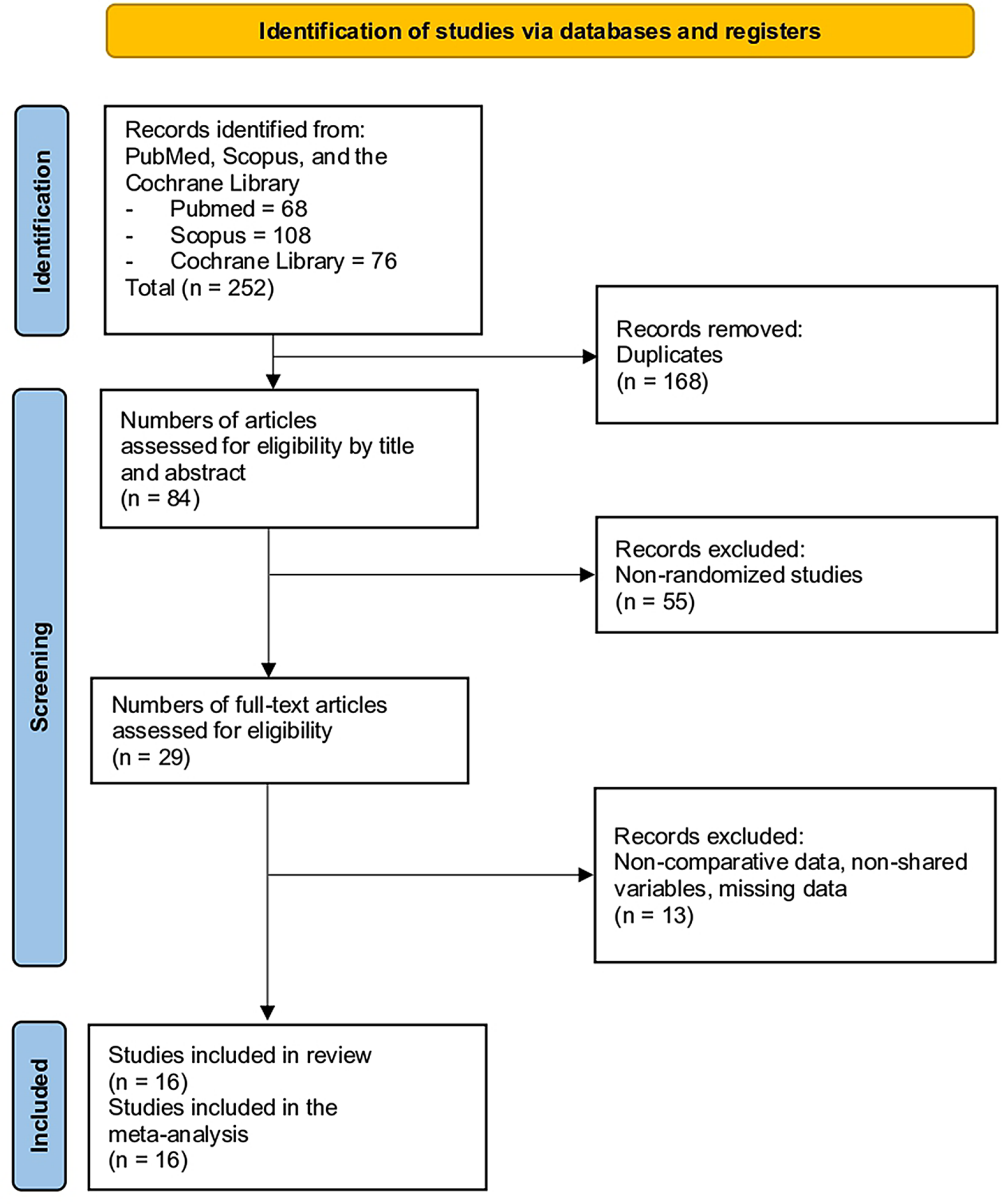
Study selection flow diagram (Preferred Reporting Items for Systematic reviews and meta-analyses)

The initial search yielded a total of 252 studies. After removing duplicates, 168 studies were eliminated, leaving 84 for further screening. After reviewing the titles and abstracts, excluding previous reviews and other nonrandomized clinical trial designs, 55 studies were removed, resulting in a total of 29 studies. A full-text review led to the exclusion of an additional 13 studies due to issues such as the use of pregabalin in both comparative groups, incomplete data, missing data, or absence of shared variables. Ultimately, 16 studies met al.l inclusion criteria and were included in the meta-analysis (Fig. 1) [9–14, 33–42].

### Baseline characteristics

The main characteristics of these studies are summarized in Table 1. Sixteen RCTs with a pool of 1766 patients were included. The mean age ranged from 59.1 to 69 years in the pregabalin group and 57.1 to 68.2 years in the other intervention groups. However, one study did not determine the exact number of female patients. The type of TJA, pregabalin dose, and other interventions are shown in Table 1. The treatment schedules of the included studies are presented in Additional Table 1.

**Table 1:** Baseline characteristics of the 16 included studies

Study	Region	Follow-up	*n* PGB/CRL/PLA	Mean age PGB/CRL/PLA	Female PGB/CRL/PLA	Type of TJA	Doses Pregabalina/Control or PLA at day	Administration TIme	COI	Funding
Buvanendran et al. 2010 [9]	USA	30 days	120/120	64/63.3	91/84	TKA	300 mg/PLA	NR	NR	NR
Carmichael et al. 2013 [33]	Canada	6 months	15/16	59.1/61.3	8/6	THA	150 mg/PLA	NR	NR	YES
Clarke et al. 2015 [10]	Canada	3 months	92/92	60.2/60.1	42/38	THA	150 mg/PLA	≤ 2 h	NR	YES
Imani et al. 2023 [11]	Iran	6 months	20/20/20	67.6/65.5/66.8	19/20/17	TKA	150 mg/60 mg duloxetine/ PLA	≤ 2 h	No	No
Jain et al. 2012 [13]	India	2 days	20/20	59.7/ 57.1	11/15	TKA	150 mg/NR	≤ 2 h	No	Yes
Kadic et al. 2016 [34]	The Netherlands	3 days	30/30	62.8/ 65.5	15/14	TKA	150mg < 65 years old or 75mg > 65 years old/PLA	NR	No	No
Lee et al. 2015 [14]	Korea	2 days	21/20	63.38/ 67.60	NR	TKA	400 mg celecoxib + 150 mg pregabalin/ 400 mg celecoxib	≤ 2 h	Yes	No
Lee et al. 2018 [35]	Korea	1 day	33/31/31	68.4/68.8/67.2	15/13 /12	TKA + THA	dexmedetomidine/150 mg + dexmedetomidine/PLA	NR	NR	NR
Lubis et al. 2018 [36]	Indonesia	3 days	10/10/10	66.1/65.9/68.2	7/7/9	TKA	(150 mg + celecoxib 400 mg)/ three times of 150 mg + celecoxib 400 mg/PLA	> 24 h and ≤ 2 h	No	NR
Martinez et al. 2014 [37]	France-USA	2 days	35/34/35/38	64/60/59/64	15/23/17/13	THA	150 mg/ ketamin/ combination/PLA	NR	No	No
Mathiesen et al. 2008 [38]	Denmark	1 day	40/42/38	67/68/66	26/20/20	THA	300 mg/300 mg + 8 mg dexamethasone/PLA	≤ 2 h	No	Yes
Niruthisard et al. 2013 [39]	Thailand	2 days	25/23/27	69/66/67	23/22/25	TKA	150 mg/ celecoxib 400 mg/PLA	≤ 2 h	No	Yes
Singla et al. 2014 [40]	USA	6 weeks	98/96/98	63.0/63.7/63.3	60/61/54	TKA	150 mg/300 mg/PLA	8–12 h	Yes	No
YaDeau et al. 2015 [12]	USA	14 days	30/30/30/30	67/65/68/66	18/13/23/14	TKA	100/200/300 mg/PLA	≤ 2 h	NR	Yes
Yik et al. 2019 [41]	Singapure	6 months	45/42	65.1/66.6	31/29	TKA	75 mg/PLA	≤ 2 h	No	No
Zhou et al. 2023 [42]	China	2 days	38/38/37/36	64.9/61.5/63.0/62.6	30/28/29/27	TKA	300 mg/ celecoxib 400 mg/combination or PLA	8–12 h	No	NR

*The control group comprised patients treated with Ketamine (3 lg.kg_1.h) or duloxetin or celecoxib (200 mg to 400 mg) or dexamethasone or dexmedetomidine (0.5 µg/ kg); COI: Conflict of interests; CRL: Control; NA: Not applicable; NR: Not reported; PGB: Pregabalin; PLA: Placebo; THA: total hip arthroplasty TKA: total knee arthroplasty

### Risk of bias

**Fig. 2:**
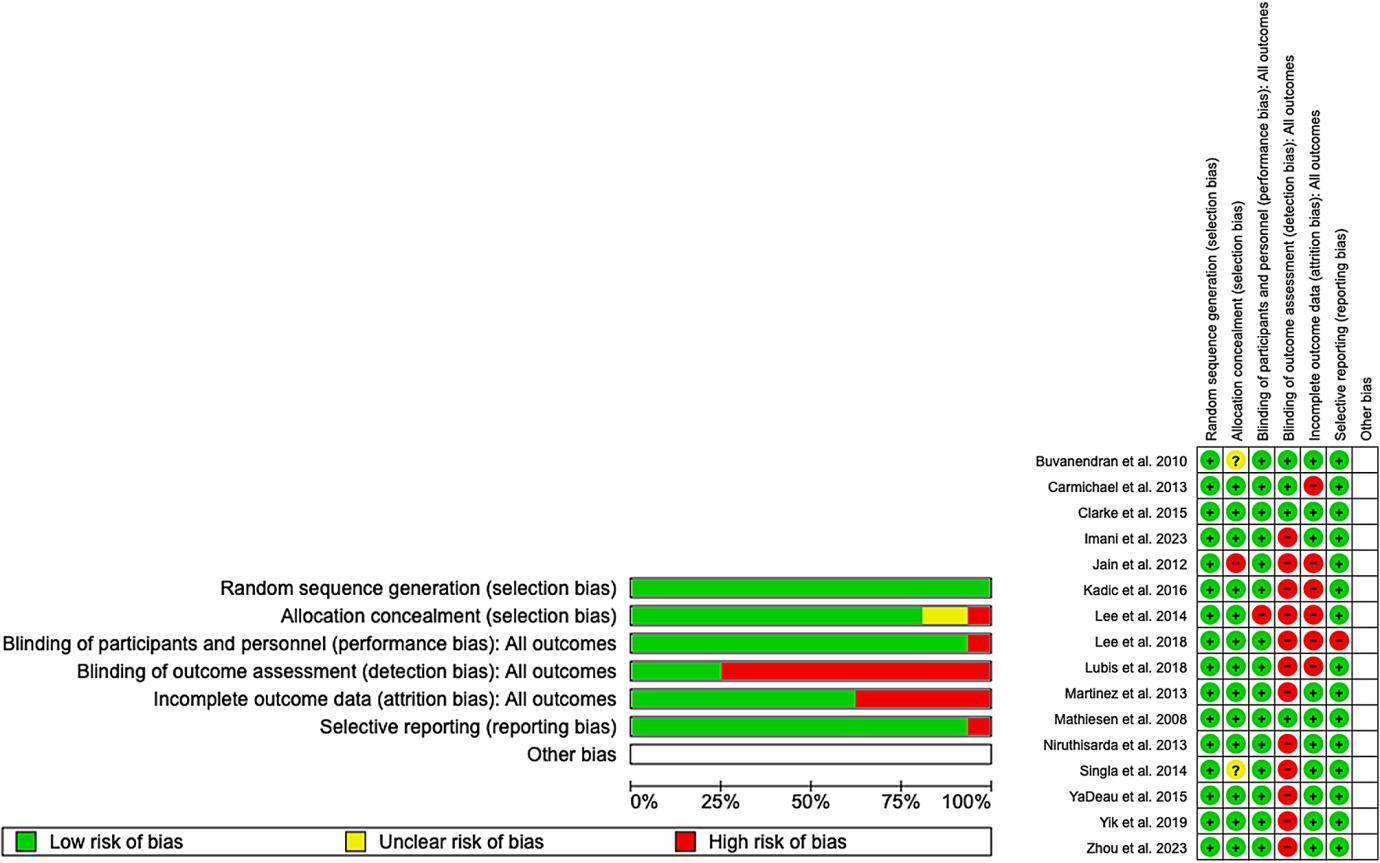
Assessment of the risk of bias (green = low risk; red = high risk; white = unknown)

The complete and individualized risks of bias in the included studies are shown in Fig. 2. An explanation of each criterion is provided in Additional File 2. These studies demonstrated a low risk of bias regarding random sequence generation, the blinding of participants and personnel, and reporting. There was a moderate risk of bias in the allocation concealment. Finally, there was a high risk of blinding the outcome assessment and incomplete outcome data.

### GRADE

The Grading of Recommendations, Assessment, Development, and Evaluation (GRADE) summary of the results of these three comparisons is shown in Table 2. In the case of VAS at 24 h, opioid consumption, and ROM (at all follow-up times), certainty was moderate or high, but VAS at 48 and 72 h and opioid consumption at 24 h and 48 h showed low or very low certainty. The studies presented a high risk of publication bias detected through funnel plots, and to a lesser extent, serious inconsistencies and indirectness.

**Table 2:** GRADE assessment of the quality of the evidence and the strength of the recommendations

Certainty assessment							№ of patients		Effect		Certainty	Importance
№ of studies	Study design	Risk of bias	Inconsistency	Indirectness	Imprecision	Other considerations	[intervention]	[comparison]	Relative (95% CI)	Absolute (95% CI)		
**VAS/NRS at 24 h**												
10	randomised trials	not serious	serious^a^	serious^b^	not serious	publication bias strongly suspected strong association dose response gradient^c^			-	MD **0.53 lower** (0.93 lower to 0.12 lower)	⨁⨁⨁◯ Moderate^a, b,c^	CRITICAL
**VAS/NRS 48 h**												
10	randomised trials	not serious	serious^a^	serious^b^	not serious	publication bias strongly suspected dose response gradient^c^			-	MD **0.58 lower** (0.93 lower to 0.22 lower)	⨁⨁◯◯ Low^a, b,c^	CRITICAL
**VAS/NRS 72 h**												
6	randomised trials	not serious	serious^a^	serious^b^	not serious	publication bias strongly suspected dose response gradient^c^			-	MD **0.66 lower** (1.1 lower to 0.22 lower)	⨁⨁◯◯ Low^a, b,c^	CRITICAL
**Opioid consumption at 24 h**												
9	randomised trials	not serious	serious^a^	serious^b^	not serious	publication bias strongly suspected dose response gradient^c^			-	SMD **0.52 SD lower** (0.8 lower to 0.24 lower)	⨁⨁◯◯ Low^a, b,c^	CRITICAL
**Opioid consumption at 48 h**												
10	randomised trials	not serious	serious^a^	serious^b^	not serious	publication bias strongly suspected^c^			-	SMD **0.74 SD lower** (1.22 lower to 0.26 lower)	⨁◯◯◯ Very low^a, b,c^	CRITICAL
**Opioid consumption at 72 h**												
3	randomised trials	not serious	not serious	not serious	not serious	publication bias strongly suspected^c^			-	SMD **1.33 SD lower** (2.16 lower to 0.49 lower)	⨁⨁⨁◯ Moderate^c^	CRITICAL
**ROM at 24 h**												
5	randomised trials	not serious	serious^a^	not serious	not serious	publication bias strongly suspected strong association dose response gradient^c^			-	SMD **0.49 SD higher** (0.39 lower to 1.38 higher)	⨁⨁⨁⨁ High^a, c^	CRITICAL
**ROM at 48 h**												
5	randomised trials	not serious	not serious	not serious	not serious	publication bias strongly suspected strong association dose response gradient^c^			-	SMD **0.68 SD higher** (0.1 lower to 1.46 higher)	⨁⨁⨁⨁ High^c^	CRITICAL
**ROM at 72 h**												
5	randomised trials	not serious	not serious	not serious	not serious	dose response gradient			-	SMD **1.11 SD higher** (0.13 higher to 2.09 higher)	⨁⨁⨁⨁ High	CRITICAL

(a) Wide variability; (b) Differences in interventions and control group; (c) Publication bias assessed using the funnel plot; CI: confidence interval; MD: mean difference; SMD: standardised mean difference

### Publication bias

Visual inspection of the funnel plot revealed asymmetry, suggesting the potential for publication bias (Additional Fig. 1 file).

### Outcomes

#### VAS assessment

**Fig. 3:**
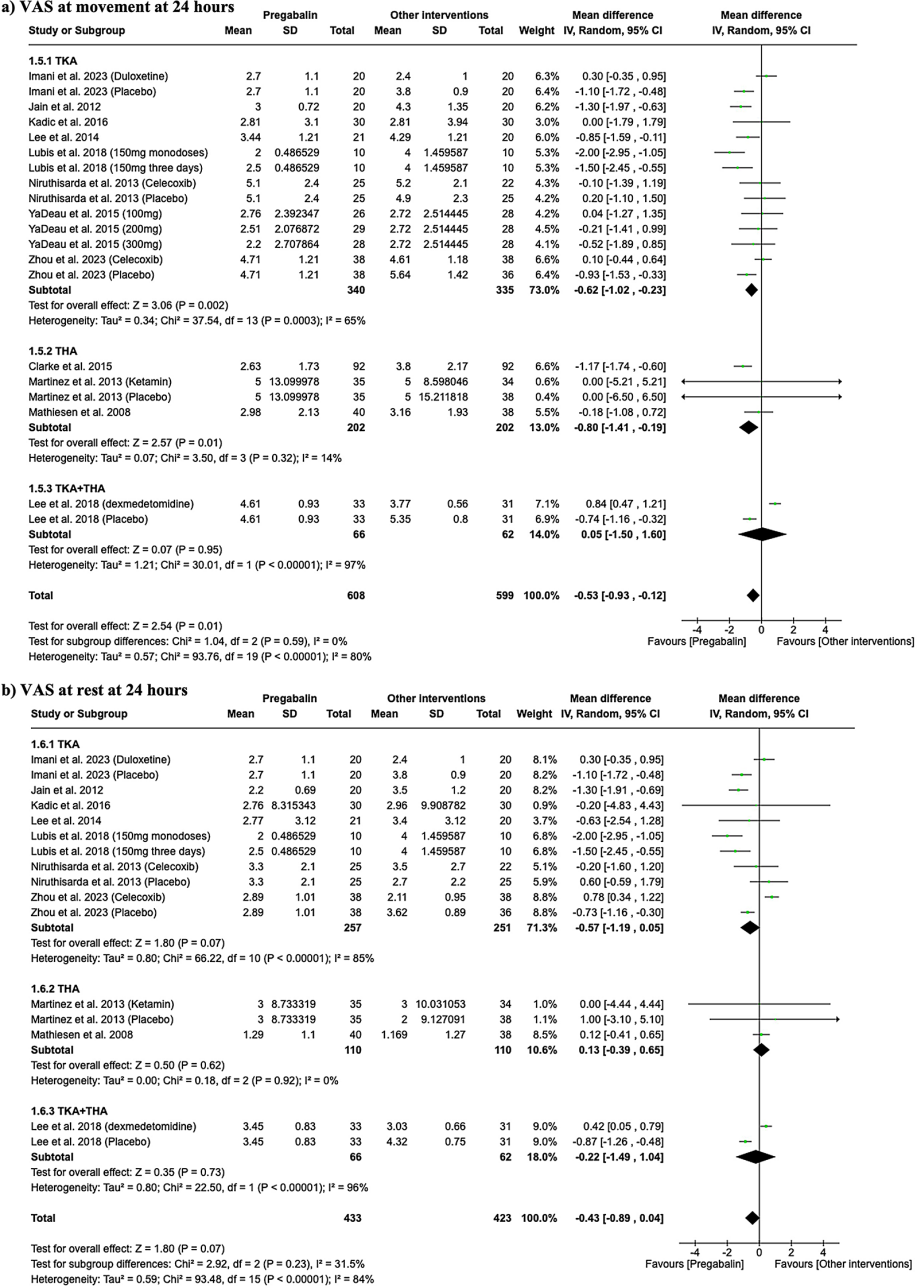
Forest plots showing VAS results at 24 h during movement (**a**) and at rest (**b**)

In the evaluation of VAS scores 24 h post-surgery during movement (Fig. 3a), significant differences were observed between pregabalin and other interventions such as celecoxib, duloxetine, or placebo. For TKA, pregabalin was associated with more effectiveness (MD -0.62, 95% CI -1.02 to -0.23; I^2^ = 65%), and in THA also was associated with greater effectiveness (MD -0.80, 95% CI -1.41 to -0.19; I^2^ = 14%). However, when the THA and TKA data were combined, no significant differences were found (MD 0.05, 95% CI -1.50 to 1.60; I^2^ = 97%;). Regarding the VAS scores at rest (Fig. 3b), no significant differences were detected across any subgroup. In TKA, the results were not significant, as well as in THA and in the combined THA/TKA group.

At 48 h post-surgery during movement, significant differences were noted with pregabalin which was associated with more effectiveness than other interventions in both TKA (MD -0.53, 95% CI -0.90 to -0.15; I^2^ = 71%) and THA (MD -1.15, 95% CI -1.77 to -0.52; I^2^ = 0%). Conversely, when assessing the VAS scores at rest, no significant differences were observed.

**Fig. 4:**
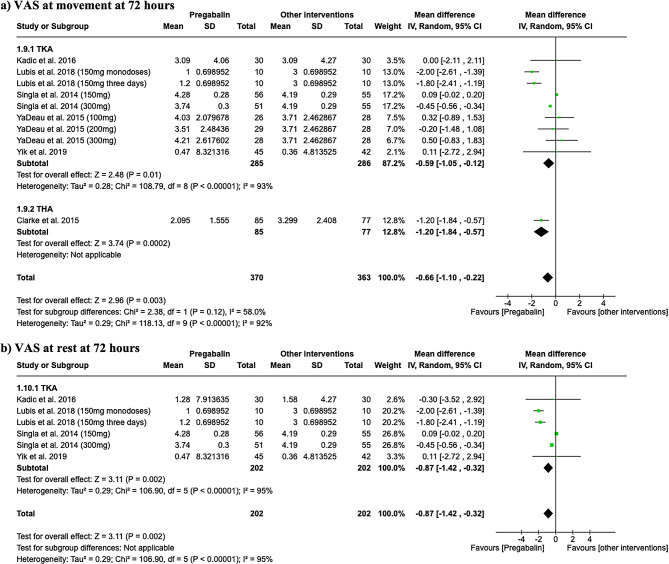
Forest plots showing pain assessed by VAS at 72 h in movement (**a**) and at rest (**b**)

At 72 h post-surgery during movement, the VAS scores for both TKA and THA subgroups showed significant differences associating pregabalin with lower pain. For TKA, the results were significant (MD -0.59, 95% CI -1.05 to -0.12), and for THA, pregabalin also was associated with significant pain reduction (MD -0.66, 95% CI -1.10 to -0.22), as depicted in Fig. 4a. Moreover, when the VAS was assessed at rest, significant differences were found in the TKA group in favor of pregabalin (MD -0.87, 95% CI -1.42 to -0.32; I^2^ = 95%), as shown in Fig. 4b.

At seven days post-surgery for patients who underwent THA, significant differences were observed, associating pregabalin with lower pain, with an MD of -1.23 (95% CI -2.09 to -0.37; I^2^ = 44%). However, it is noted that there were no studies analyzing VAS scores at seven days in TKA.

### Opioid consumption

**Fig. 5:**
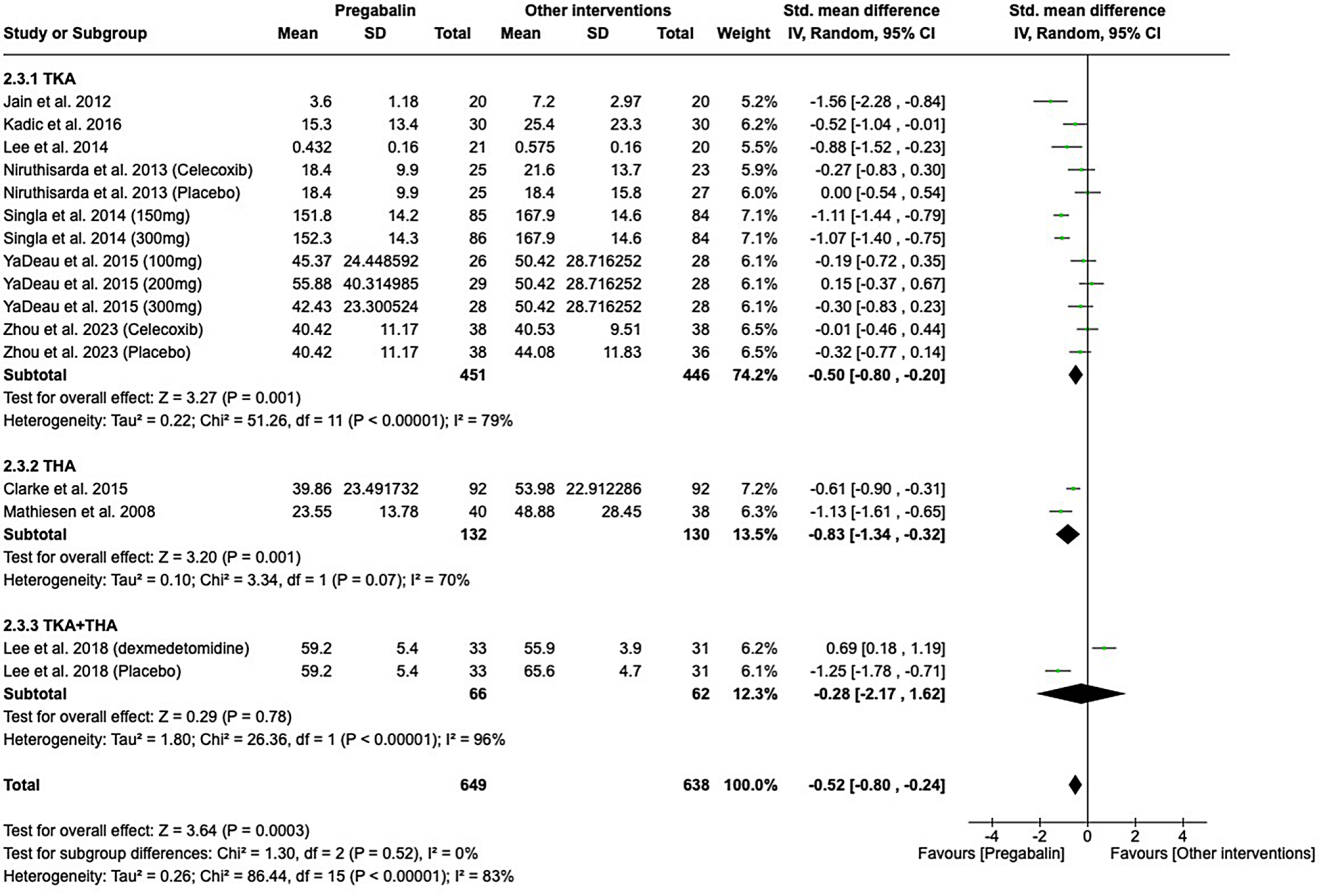
Forest plot showing opioid consumption at 24 h

**Fig. 6:**
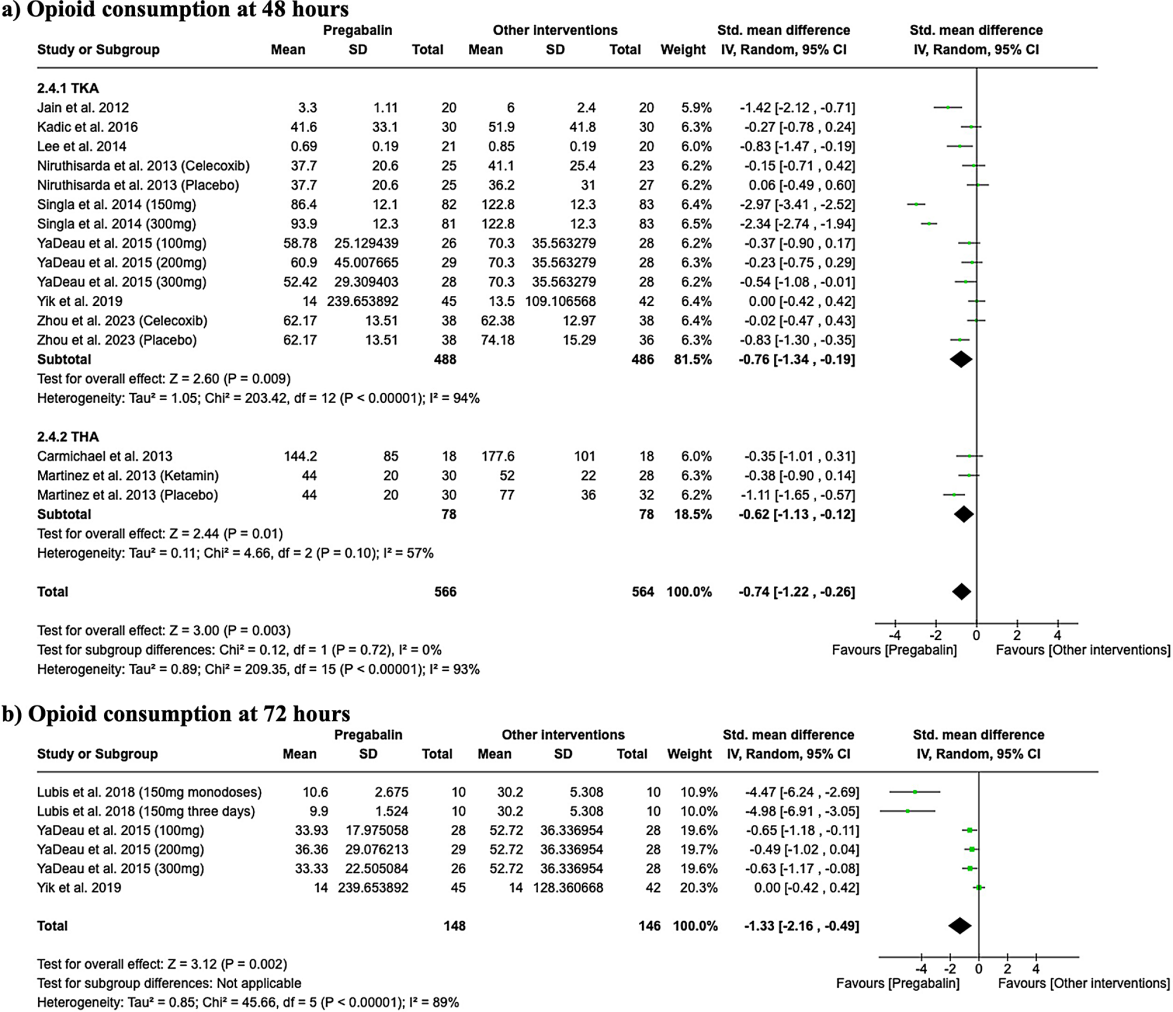
Forest plots showing opioid consumption at 48 h (**a**) and 72 h (**b**)

At 24 h post-surgery, exposure to pregabalin was associated with reduced opioid consumption in both the TKA and THA subgroups. Specifically, in the TKA subgroup, the standardized mean difference (SMD) was − 0.50 (95% CI -0.80 to -0.20; I2 = 79%), and in the THA subgroup, the SMD was − 0.83 (95% CI -1.34 to -0.32; I2 = 70%), as shown in Fig. 5. At 48 h post-surgery, reduced opioid consumption was associated with pregabalin exposure. In the TKA subgroup, the SMD was − 0.76 (95% CI -1.34 to -0.19; I2 = 94%), and in the THA subgroup, the SMD was − 0.62 (95% CI -1.13 to -0.12; I2 = 57%), as depicted in Fig. 6a. At 72 h, a further reduction in opioid consumption was associated with pregabalin exposure in the TKA subgroup, with an SMD of -1.33 (95% CI -2.16 to -0.49; I2 = 89%), as illustrated in Fig. 6b.

### Range of motion

**Fig. 7:**
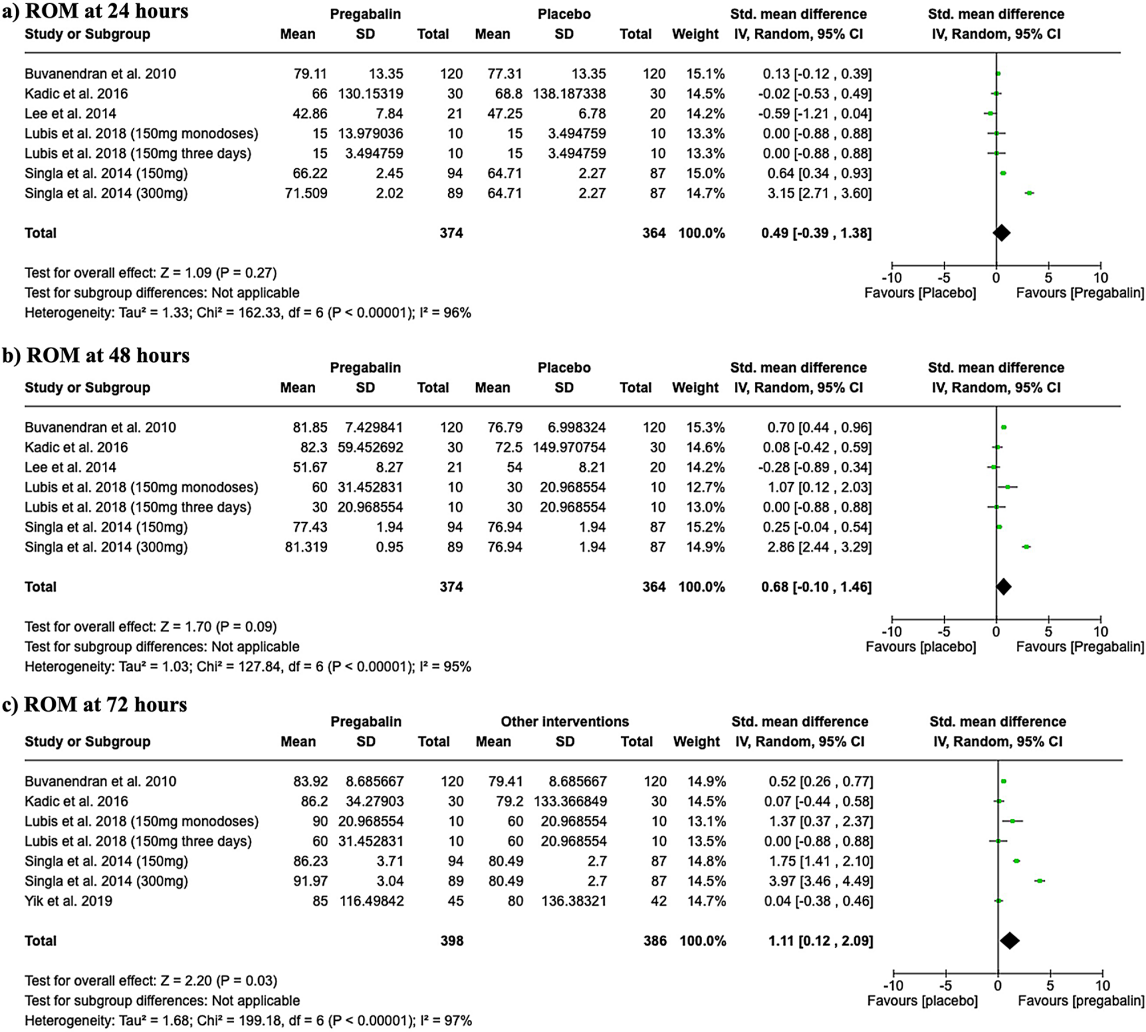
Forest plots showing the degree of knee flexion in patients undergoing TKA measured in six studies at 24 h (**a**), 48 h (**b**), and 72 h (**c**)

The degree of knee flexion in patients undergoing TKA was measured in six studies. At 24 h post-surgery, there were no significant differences in range of motion (ROM) between the pregabalin group and placebo, as illustrated in Fig. 7a. Similarly, at 48 h, no significant differences were observed, as shown in Fig. 7b. However, at 72 h post-surgery, pregabalin exposure was associated with a significant increase in ROM compared to the placebo group, with a standardized mean difference (SMD) of 1.11 (95% CI 0.12 to 2.09; I2 = 97%), as depicted in Fig. 7c.

### Adverse events

Pregabalin was associated with a significant increase in sedation at 24 h post-TKA surgery, with an odds ratio (OR) of 2.27 (95% CI 1.13 to 4.56; participants = 216), and at 48 h post-TKA, the OR was 2.54 (95% CI 1.11 to 5.79; participants = 405; I^2^ = 0) compared to controls. Additionally, pregabalin was significantly associated with reduction of nausea, with an OR of 0.30 (95% CI 0.09 to 0.99; participants = 142; I^2^ = 0) and vomiting at 48 h post-TKA, with an OR of 0.17 (95% CI 0.04 to 0.65; participants = 246; I^2^ = 0). Furthermore, pregabalin was related to an increase of the incidence of diplopia and blurred vision at 24 h in the combined TKA and THA subgroups (OR 4.68, 95% CI 1.37 to 15.99; participants = 95; I^2^ = 34), and specifically in the TKA subgroup (OR 9.00, 95% CI 1.12 to 72.36; participants = 276; I^2^ = 0). No significant differences were observed in the remaining adverse events as detailed in Table 3.

**Table 3:** Adverse events assessment

Effect size	*n* participants	Random/Fixed effect model (OR 95% CI)	I^2^ (%)	*P*-Value
**Sedation**				
TKA at 24 h	216	OR 2.27, 95% CI 1.13 to 4.56	N/A	0.02
TKA at 48 h	405	OR 2.54, 95% CI 1.11 to 5.79	0	0.03
THA at 24 h	114	OR 1.11, 95% CI 0.51 to 2.41	7	0.80
THA at 48 h	142	OR 1.80, 95% CI 0.75 to 4.32	0	0.19
**Dizziness**				
TKA at 24 h	276	OR 1.87, 95% CI 1.00 to 3.50	0	0.05
TKA at 48 h	358	OR 1.42, 95% CI 0.61 to 3.29	0	0.41
THA at 24 h	36	OR 5.09, 95% CI 0.89 to 29.27	N/A	0.07
THA at 48 h	142	OR 2.99, 95% CI 0.22 to 41.25	66	0.41
TKA + THA at 24 h	95	OR 1.05, 95% CI 0.39 to 2.82	0	0.93
**Nausea**				
TKA at 24 h	332	OR 1.46, 95% CI 0.81 to 2.61	29	0.21
TKA at 48 h	246	OR 0.50, 95% CI 0.18 to 1.38	30	0.18
THA at 24 h	142	OR 0.30, 95% CI 0.09 to 0.99	0	0.05
TKA + THA at 24 h	98	OR 1.17, 95% CI 0.45 to 3.03	0	0.75
**Vomiting**				
TKA at 24 h	216	OR 0.68, 95% CI 0.19 to 2.48	N/A	0.56
TKA at 48 h	246	OR 0.17, 95% CI 0.04 to 0.65	0	0.01
THA at 24 h	78	OR 1.78, 95% CI 0.58 to 5.49	N/A	0.32
THA at 48 h	142	OR 0.67, 95% CI 0.18 to 2.51	0	0.55
TKA + THA at 24 h	95	OR 0.81, 95% CI 0.29to 2.29	0	0.69
**PONV**				
TKA at 24 h	60	OR 2.25, 95% CI 0.51 to 9.99	N/A	0.29
TKA at 48 h	150	OR 0.65, 95% CI 0.27 to 1.57	0	0.33
THA at 48 h	142	OR 1.03, 95% CI 0.43 to 2.48	0	0.98
**Diplopia and blurred vision at 24 h**				
TKA	276	OR 9.00, 95% CI 1.12 to 72.36]	0	0.04
THA	36	OR 3.17, 95% CI 0.12 to 83.17	N/A	0.49
THA + TKA	95	OR 4.68, 95% CI 1.37 to 15.99	34	0.01
**Dry mouth**				
TKA at 24 h	216	OR 7.71, 95% CI 0.93 to 63.75	N/A	0.06
TKA at 48 h	246	OR 0.84, 95% CI 0.16 to 4.37	52	0.84
THA + TKA at 24 h	95	OR 1.55, 95% CI 0.32 to 7.51	60	0.58
**Headache**				
TKA at 24 h	216	OR 7.47, 95% CI 0.38 to 146.44	N/A	0.19
TKA at 48 h	206	OR 3.21, 95% CI 0.13 to 79.75	N/A	0.48
THA + TKA at 24 h	95	OR 2.59, 95% CI 0.77 to 8.76	0	0.13
**Other gastrointestinal events at 48 h**				
TKA	152	OR 0.33, 95% CI 0.09 to 1.27	N/A	0.11
**Urine retention at 48 h**				
TKA	152	OR 2.31, 95% CI 0.69 to 7.75	6	0.18
THA	142	OR 1.24, 95% CI 0.53 to 2.91	0	0.62

* CI: confidence interval; N/A: Not applicable; OR: Odds Ratio; THA: total hip arthroplasty TKA: total knee arthroplasty

### Analysis of patient discontinuation rates

Patients excluded for adverse events or inadequate pain control were included in four studies of patients who underwent THA. There were no differences between the pregabalin group and other interventions (Additional Fig. 2a file). Nine studies excluded patients for any cause. In this case, there were no significant differences between groups either in the case of patients undergoing TKA or THA or in studies where the type of surgery was not differentiated (Additional Fig. 2b file).

### Impact of timing of pregabalin administration

Table 4 presents the results according to the timing of pregabalin administration on VAS score, opioid consumption, and ROM outcomes. Administration of pregabalin for > 24 h before surgery resulted in a significant association with the reduction of pain level, as assessed by the Visual Analog Scale (VAS) at 72 h, both during movement and at rest (*p* = 0.0004). Similarly, the administration of pregabalin for > 24 h prior to surgery also was associated with a significant reduction in opioid consumption at 72 h (*p* < 0.0001).

**Table 4:** Investigation of sensitivity according to induction time of Pregabalin on VAS, opioid consumption and ROM outcomes

Effect size	*n* participants	Random/Fixed effect model (MD 95% CI; SMD 95% CI)	I^2^ (%)	*P*-Value
**VAS 24 h movement**				
More than 24 h before surgery	20	MD -1.50, 95%CI -2.45 to -0.55	--	0.22
8–12 h before surgery	112	MD -0.41, 95%CI -1.41to 0.60	84	
2 or less hours before surgery	713	MD -0.65, 95%CI -1.05 to -0.25	56	
**VAS 24 h rest**				
More than 24 h before surgery	20	MD -1.50, 95%CI -2.45 to -0.55	----	0.13
8–12 h before surgery	112	MD 0.02, 95%CI -1.46 to 1.50	96	
2 or less hours before surgery	418	MD -0.50, 95%CI -1.12 to 0.11	74	
**VAS 48 h movement**				
More than 24 h before surgery	20	MD -1.00, 95%CI -1.97 to -0.03	---	0.10
8–12 h before surgery	112	MD -0.13, 95%CI -0.55 to 0.30	53	
2 or less hours before surgery	588	MD -0.71, 95%CI -1.17 to -0.24	61	
**VAS 48 h rest**				
More than 24 h before surgery	20	MD -1.00, 95%CI -1.97 to -0.03	---	0.23
8–12 h before surgery	112	MD -0.15, 95%CI -0.47 to 0.18	2	
2 or less hours before surgery	427	MD 0.01, 95%CI -0.84 to 0.86	85	
**VAS 72 h movement**				
More than 24 h before surgery	20	MD -1.80, 95%CI -2.41 to -1.19	---	0.0004
8–12 h before surgery	162	MD -0.18, 95%CI -0.71 to 0.35	98	
2 or less hours before surgery	380	MD -0.59, 95%CI -1.49 to 0.30	55	
**VAS 72 h rest**				
More than 24 h before surgery	20	MD -1.80, 95%CI -2.41 to -1.19	----	0.0004
8–12 h before surgery	162	MD -0.18, 95%CI -0.71 to 0.35	98	
2 or less hours before surgery	107	MD -1.42, 95%CI -3.27 to 0.43	51	
**Opioid consumption at 24 h**				
More than 24 h before surgery	------	-------	-----	0.58
8–12 h before surgery	367	SMD − 0.65, 95%CI -1.17 to -0.13	87	
2 or less hours before surgery	624	SMD − 0.47, 95%CI -0.84 to -0.10	82	
**Opioid consumption at 48 h**				
More than 24 h before surgery	36	SMD − 0.35, 95%CI -1.01 to 0.31	----	0.23
8–12 h before surgery	367	SMD − 1.54, 95%CI -2.86 to -0.22	97	
2 or less hours before surgery	111	SMD − 0.36, 95%CI -0.69 to -0.04	75	
**Opioid consumption at 72 h**				
More than 24 h before surgery	20	SMD − 4.98, 95%CI -6.91 to -3.05	-----	< 0.0001
8–12 h before surgery	------	-------	-----	
2 or less hours before surgery	218	SMD − 0.81, 95%CI -1.48 to -0.14	84	
**ROM at 24 h**				
More than 24 h before surgery	20	SMD 0.00, 95%CI -0.88 to 0.88	---	0.30
8–12 h before surgery	270	SMD 1.89, 95%CI -0.58 to 4.36	99	
2 or less hours before surgery	301	SMD − 0.10, 95%CI -0.57 to 0.37	54	
**ROM at 48 h**				
More than 24 h before surgery	20	SMD 0.00, 95%CI -0.88 to 0.88	---	0.46
8–12 h before surgery	270	SMD 1.55, 95%CI -1.01 to 4.11	99	
2 or less hours before surgery	301	SMD 0.47, 95%CI -0.25 to 1.18	78	
**ROM at 72 h**				
More than 24 h before surgery	20	SMD 0.00, 95%CI -0.88 to 0.88	---	0.06
8–12 h before surgery	270	SMD 2.85, 95%CI 0.68 to 5.03	98	
2 or less hours before surgery	347	SMD 0.49, 95%CI -0.03 to 1.00	72	

* CI: confidence interval; MD: mean difference; SMD: standardized mean difference; THA: total hip arthroplasty TKA: total knee arthroplasty

## Discussion

In the current study, pregabalin was effectively associated with a reduction in postoperative pain, as assessed using the VAS at 24 h during movement, 48 h at rest, 72 h under both conditions, and 7 days after the procedure. Although ROM showed no improvement at 24–48 h, pregabalin was associated with an improvement in ROM at 72 h. Additionally, pregabalin was associated with decreased opioid consumption at 24, 48, and 72 h. Medication also increased the incidence of sedation but decreased the incidence of nausea and vomiting.

Pregabalin has been shown to significantly reduce opioid consumption in the first 24, 48, and 72 h post-surgery in both total knee arthroplasty and total hip arthroplasty. This finding is particularly significant in the context of the current opioid crisis, in which there is an intense need for safe and effective alternatives to postoperative pain management. The ability of pregabalin to decrease the need for opioids is attributed to its mechanism of action, which modulates pain transmission in the central nervous system by reducing the release of excitatory neurotransmitters. This not only improves pain control but also minimizes the adverse effects associated with high doses of opioids, such as respiratory depression, constipation, dependence, and the risk of overdose. Additionally, some studies have administered pregabalin before surgery, which is a promising strategy. Preoperative pain management with opioids has shown a tendency to increase opioid consumption postoperatively with a corresponding risk of dependence and other adverse events. The prolonged use of postoperative opioids has also been associated with anxiety and depression [43], which reinforces the need to explore alternative pain control strategies, such as the multimodal approach [44].

In our study, the analysis of the range of motion, specifically knee flexion, was only performed in patients who underwent TKA. At 24 and 48 h, no significant differences were observed. However, at three days postoperatively, the group treated with pregabalin was associated with a significant improvement in range of motion compared to the untreated group, with a notable difference of almost 10 degrees. This physiological improvement may be due to its effects in reducing pain, local inflammation, and muscle spasticity through its influence on the central and peripheral nerves [8, 45, 46]. Effective postoperative pain control is directly related to an improvement in early mobility, which is crucial for preventing postoperative complications, reducing the duration of hospital stay, and favorably impacting costs [47]. It has been observed that regular pain control is a critical factor in the failure of same-day discharge after a total hip arthroplasty, suggesting the use of pregabalin or local infiltrations as viable alternatives [48, 49]. Early discharge and mobilization are related to greater patient satisfaction [50, 51], which is an important indicator of healthcare quality [52].

Pregabalin in cases of total knee arthroplasty showed an increased rate of sedation, a finding highlighted only by Buvanendran et al. [9], who reported an increase in postoperative sedation and confusion. This phenomenon could be attributed to the abrupt onset of pregabalin use, as this was one of the first trials in this area [9]. Additionally, the incidence of vomiting was lower with pregabalin in patients who underwent TKA within the first 48 h. There were no significant differences in treatment discontinuation due to adverse events or any other cause, suggesting an adequate safety profile for pregabalin, with generally mild adverse events. The rest of the adverse events showed no significant differences.

Owing to the small number of included studies, this study was unable to analyze the influence of dosage on safety or efficacy, nor could it establish the optimal dose. A meta-analysis indicated that 300 mg pregabalin, the highest dose used in our study, had the best safety profile in patients with fibromyalgia [53]. Nearly all studies used a daily dose of 150 mg, which precluded a formal analysis of varying pregabalin dosages. However, it was possible to analyze the timing of pregabalin administration. The results indicated that initiating pregabalin at least one day to two weeks prior to surgery could be beneficial both for reducing postoperative pain and decreasing opioid consumption within the first 72 h. These results are consistent with those of Buvanendran et al., who observed that starting treatment up to two weeks before the procedure could improve adherence to pregabalin [9].

Although this meta-analysis primarily focused on examining the isolated effect of pregabalin, there were studies that included an additional arm combining pregabalin with other drugs such as celecoxib, ketamine, dexamethasone, or dexmedetomidine. This combination enhanced postoperative pain management and yielded satisfactory results in the analysis of pain levels and opioid consumption. These findings underscore the importance of multimodal pain management that employs various mechanisms of action to optimize patient outcomes. This approach highlights the potential benefits of integrating different pharmacological strategies to achieve superior control of postoperative pain and reduce reliance on opioids.

In the current meta-analysis, a significantly larger number of studies have been included compared to previous reviews, which enhances the robustness of the results, especially focusing on pregabalin, and extends the evaluation of opioid consumption up to 72 h compared to 48 h in previous analyses, confirming a significant reduction in opioid consumption with pregabalin. However, Mao et al. observed that the VAS pain score was not significantly reduced [22]. In contrast, a 2016 meta-analysis focused solely on pregabalin and noted a significant reduction in pain and morphine consumption at 24 and 48 h, although it reported an increase in dizziness and sedation and a reduction in nausea and vomiting [23]. However, our study did not show differences in almost all adverse events, possibly due to the accuracy of data collection timing, unlike other studies with mixed follow-ups. Dong et al. highlighted that multiple doses of pregabalin reduced pain more than a single doses [23].

Han et al. reported a higher incidence of constipation and itching with pregabalin [25]. In contrast, Li et al. observed greater benefits in the THA subgroups in terms of reduced opioid consumption and pain at rest at 72 houirs [26]. Our analysis also stands out for including a greater number of adverse events and for differentiating between the types of surgical procedures (THA or TKA). Finally, Hamilton et al. observed no differences in efficacy in a meta-analysis that used gabapentinoids in general without differentiating between them [24].

### Limitations

This study has several limitations that should be considered when interpreting the results. First, formal publication bias tests, such as the Egger test, could not be used due to limitations of the software employed. Additionally, the representativeness of some subgroups within the sample was limited by the low number of available studies that specifically focused on these populations. Also, future studies should report the percentage of patients who achieved or did not achieve the minimal clinically important difference (MCID). This approach would allow for a more comprehensive evaluation of the clinical relevance of these findings [54]. Furthermore, uniformity in reporting adverse events was poor, with varying times for recording these events across different studies. Moreover, this inconsistency was exacerbated by the limited number of articles available on each safety variable. Another limitation is that the main variables were evaluated in the short term. Additionally, the studies did not include reports on the use of postoperative local infiltration with anti-inflammatory or anesthetic agents nor did they control for confounding variables such as different therapeutic regimens. Therefore, these studies did not provide adjusted data that would allow for a more precise analysis adjusted for potential confounders. Finally, there were inconsistencies in the reporting of other functionality scales that could have provided additional comparative data.

## Conclusions

Pregabalin, administered prior to surgery and after the surgery, was associated with a significantly reduction of postoperative pain during the first three days after total joint arthroplasty, significantly decreased opioid consumption, and enhanced range of motion at three days post-surgery, with a favorable safety profile.

## Electronic supplementary material

Below is the link to the electronic supplementary material.

Supplementary Material 1: Additional file 1: Word document of Pubmed search strategy

Supplementary Material 2: Additional file 2: Word document of Risk of bias judgement

Supplementary Material 3: Additional Table 1 file: word document of treatment schemes of the included studies.

Supplementary Material 4: Additional Fig. 1 file: JPG file showing asymmetry of the funnel plot

Supplementary Material 5: Additional Fig. 2 file: JPG file of adverse events

## Data Availability

No datasets were generated or analysed during the current study.
