# Supplementary material for: Efficacy and safety of pregabalin for postoperative pain after total hip and knee arthroplasty: a systematic review and meta-analysis
Source: J Orthop Surg Res. 2025 Mar 11;20:261. doi: 10.1186/s13018-025-05675-6 (PMC11895303; doi:10.1186/s13018-025-05675-6)
Supplement: Supplementary file 1 — Supplementary Material 1: Additional file 1: Word document of Pubmed search strategy [file 13018_2025_5675_MOESM1_ESM.docx]

**Additional File 1.** Pubmed search strategy.

Search: **(Pregabalin OR lyrica) AND ("total joint arthroplasty" OR TJA OR "arthroplasty" OR "knee arthoplasty" OR "hip arthroplasty" OR "hip replacement" OR "knee replacement" OR "joint replacement" OR TKA OR THA)**

("pregabalin"[MeSH Terms] OR "pregabalin"[All Fields] OR "pregabalin s"[All Fields] OR "pregabaline"[All Fields] OR ("pregabalin"[MeSH Terms] OR "pregabalin"[All Fields] OR "lyrica"[All Fields] OR "pregabalin s"[All Fields] OR "pregabaline"[All Fields])) AND ("total joint arthroplasty"[All Fields] OR "TJA"[All Fields] OR "arthroplasty"[All Fields] OR "knee arthoplasty"[All Fields] OR "hip arthroplasty"[All Fields] OR "hip replacement"[All Fields] OR "knee replacement"[All Fields] OR "joint replacement"[All Fields] OR "TKA"[All Fields] OR "THA"[All Fields])

**Translations**

**Pregabalin:** "pregabalin"[MeSH Terms] OR "pregabalin"[All Fields] OR "pregabalin's"[All Fields] OR "pregabaline"[All Fields]

**lyrica:** "pregabalin"[MeSH Terms] OR "pregabalin"[All Fields] OR "lyrica"[All Fields] OR "pregabalin's"[All Fields] OR "pregabaline"[All Fields]
