# Supplementary material for: Efficacy and safety of pregabalin for postoperative pain after total hip and knee arthroplasty: a systematic review and meta-analysis
Source: J Orthop Surg Res. 2025 Mar 11;20:261. doi: 10.1186/s13018-025-05675-6 (PMC11895303; doi:10.1186/s13018-025-05675-6)
Supplement: Supplementary file 2 — Supplementary Material 2: Additional file 2: Word document of Risk of bias judgement [file 13018_2025_5675_MOESM2_ESM.docx]

**Supplementary File 2.** Risk of bias judgement.

Buvanendran et al. 2010

1. Patients were randomized to a treatment group using a computer-generated randomization sequence
2. It is not indicated whether the use of this randomization system guarantees concealment of treatment allocation.
3. The physicians and nurses managing the patient perioperatively, the personnel involved with postoperative pain assessments and management of the epidural infusion, physical therapists, and the study patients were blinded to group assignments.
4. Patients were evaluated in a blinded fashion.
5. Figure 1.
6. Different kind of outcomes with complete data.

Carmichael et al. 2013

1. A computer-generated randomization schedule was used to assign patients to one of two treatment groups.
2. An interim analysis of the primary outcome measure (6 min walk test) based on the 31 patients who had completed the study was performed by a statistician who was blinded to the treatment allocation.
3. Pregabalin, celecoxib and placebo medications were prepared and dispensed by the Sunnybrook Health Sciences Centre Investigational Pharmacy (Toronto, Ontario) to maintain doubleblinded conditions. Data analyses were performed by an independent blinded statistician.
4. Data analyses were performed by an independent blinded statistician.
5. Not reported
6. Different kind of outcomes with complete data.

Clarke et al. 2015

1. A computer-generated randomization schedule was used to assign patients at random, in blocks of six, to one of the two treatment groups.
2. Patients received either pregabalin 75 mg BID or placebo BID according to the preoperative randomization allocation, starting 8 h after the preoperative dose.
3. Researchers were also blind to drug assignment during data analysis.
4. Blinding was maintained throughout the study until the code was broken upon the completion of our statistical analysis..
5. Given a repeated-measures study design, the likelihood of missing data, correlated errors within individuals, and heterogeneity among occasion variances (i.e. over time),we applied generalized estimating equations to test for differences in the timed get-upand-go test, the stair test, and the 6 min walk test between the groups.
6. All kind of outcomes.

Imani et al. 2023

1. Patients were divided into three equal groups (n=20) according to the block randomization method
2. Allocating patients to the studied groups was done by a blinded collaborator not involved in study procedures.
3. Patients and clinical researchers were also blinded to the groups and interventions.
4. Not reported
5. Figure 1.
6. Different kind of outcomes with complete data.

Jain et al. 2012

1. Patients were randomly assigned to one of the two treatment groups using a computer generated random number list.
2. Not reported
3. Both the staff nurse and the patient were not aware of the contents of the drug.
4. Not reported
5. Not reported
6. All kind of outcomes.

Kadic et al. 2016

1. The Clinical Pharmacology Department randomized the patients and supplied the coded study medication.
2. Figure 1.
3. Not a single person from the Department of Anesthesiology or the Orthopedics knew what medication was administered to any individual patient.
4. Not reported
5. Not reported
6. All kind of outcomes.

Lee et al. 2014

1. Patients were randomized to either a control or a study group by the random drawing of numbers by a personnel not involved in this study..
2. Figure 1.
3. Not reported
4. Not reported.
5. Not reported
6. Different kind of outcomes with complete data.

Lee et al. 2018

1. A physician who was not involved in the perioperative evaluation administered the capsule according to the randomization sequence
2. Figure 1.
3. The patient and the anesthesiologist were both blinded to treatment and all data were recorded by an anesthesiologist blinded to study group allocation.
4. Not reported.
5. Not reported
6. Different kind of outcomes with complete data.

Lubis et al. 2018

1. This study used block randomization with the size of 3
   according to each group.
2. Allocation proceeds by randomly selecting one of the orderings and assigning the next block of participants to study groups according to the specified sequence.
3. The double-blind design in this study included blinding of orthopedic surgeon, anesthetic operator, medical team giving the drugs, and the  patients.
4. Not reported.
5. Not reported
6. Different kind of outcomes with complete data.

Martinez et al. 2013

1. Computer-generated randomisation (Excel; Microsoft Office 2007) was based on blocks of four patients.
2. Allocations were concealed in sequentially numbered
   sealed opaque envelopes, which were opened the day
   before surgery after patients had consented to the trial.
3. None of the other investigators involved in patient management or data collection was aware of the group assignment.
4. Not reported.
5. Figure 1
6. Different kind of outcomes with complete data.

Mathiesen et al. 2008

1. Consecutive numbers according to a computer-generated block randomization schedule prepared by the hospital pharmacy.
2. Patients were randomly allocated to one of the following three treatment groups.
3. No person was aware of group assignment until all patients had been included and assessments were completed..
4. No person was aware of group assignment until all patients had been included and assessments were completed.
5. Figure 1
6. All kind of outcomes with complete data.

Niruthisarda et al. 2013

1. The randomization was done by random
   number table.
2. We allocated the patients sequentially.
3. Single-center, prospective, randomized, double-blind, placebo controlled study.
4. Not reported.
5. Figure 1
6. All kind of outcomes with complete data.

Singla et al. 2014

1. Patients were assigned a subject identification number at screening and a separate number at randomization to identify which treatment was to be received.
2. It is not clear.
3. In all three trials, investigators used the sponsor’s inter­active response technology system (via phone or Internet) to screen, randomize, and assign treatment to patients in a double-blinded manner.
4. Not reported.
5. Figure 2
6. Wide type of results.

YaDeau et al. 2015

1. The computer-generated randomization table was prepared by a research assistant not otherwise involved in the study.
2. Figure 1
3. Double-blind trial.
4. Not reported.
5. Figure 1
6. All kind of outcomes.

Yik et al. 2019

1. The recruited subjects were randomised using randomization.com, in blocks of 10.
2. Figure 1
3. Double-blind trial.
4. Not reported.
5. Figure 1
6. Different kind of outcomes.

Zhou et al. 2023

1. An assistant from the Clinical Research Center used a random number table to divide the patients into four groups.
2. Figure 1
3. double-blind randomized clinical trial
4. Not reported.
5. Figure 1
6. All types of completed outcomes.
