# Supplementary material for: Efficacy and safety of pregabalin for postoperative pain after total hip and knee arthroplasty: a systematic review and meta-analysis
Source: J Orthop Surg Res. 2025 Mar 11;20:261. doi: 10.1186/s13018-025-05675-6 (PMC11895303; doi:10.1186/s13018-025-05675-6)
Supplement: Supplementary file 3 — Supplementary Material 3: Additional Table 1 file: word document of treatment schemes of the included studies. [file 13018_2025_5675_MOESM3_ESM.docx]

**Additional Table 1.** Treatment schemes of the included studies.

| **Study** | **Treatment schemes** |
| --- | --- |
| **Buvanendran et al. 2010 [9]** | Patients randomized to the experimental arm of the study received pregabalin 300 mg orally (per os [PO]), 1–2 h before surgery, 150 mg twice daily for the first 10 postoperative days, 75 mg twice daily on Days 11 and 12, and 50 mg twice daily on Days 13 and 14. |
| **Carmichael et al. 2013 [33]** | Group 1 received pregabalin (75 mg twice per day) and celecoxib (100 mg twice per day) for two weeks before surgery and for three weeks from the day of discharge. Group 2 received identical placebo tablets for the same period of time |
| **Clarke et al. 2015 [10]** | Patients were randomly assigned to receive neither pregabalin 150 mg p.o. or placebo p.o. at the same time they received the celecoxib (2 hours before surgery).Patients received either pregabalin 75 mg BID or placebo BID according to the preoperative randomization allocation, starting 8 h after the preoperative dose and continuing throughout their hospital stay and for 7 days after discharge. |
| **Imani et al. 2023 [11]** | Group A received pregabalin 75 mg, group B duloxetine 30 mg, and group C placebo. Intervention (prescription of drugs) was performed in all groups three times, 90 minutes before surgery, then 12 and 24 hours after surgery. |
| **Jain et al. 2012 [13]** | The test drug group received pregabalin 75 mg tablets while the control group received matching placebo. Study medication was given orally twice a day, with sips of water, starting 2 h preoperatively, And during two days after surgery. |
| **Kadic et al. 2016 [34]** | Premedication consisted of two 500 mg paracetamol tablets (total 1000 mg) for all patients and of two capsules of 75 mg pregabalin (total 150 mg) or identical placebo capsules. The dose of pregabalin or placebo was reduced to one capsule ASA III patients and in patients >65 years of age. Oral study medication (identical pregabalin or placebo capsules) was continued twice daily from the operation day to the 3^rd^ postoperative day. |
| **Lee et al. 2015 [14]** | Control group received 400 mg celecoxib, and those randomized to the study group received 400 mg celecoxib plus 150 mg pregabalin approximately 1 hour prior to operation. |
| **Lee et al. 2018 [35]** | One hour before spinal anesthesia, patients received 150 mg of pregabalin or capsules containing placebo orally, and a bolus dose of 0.5 μg/ kg of intravenous dexmedetomidine was given over 10 minutes before induction of spinal anesthesia. This was followed by a continuous infusion of 0.5 μg/kg/h or the same calculated volume of normal saline until completion of the surgery. Group C received placebo pregabalin and placebo dexmedetomidine, group P received pre­gabalin and placebo dexmedetomidine, group PD received pregabalin and dexme­detomidine, and group D received placebo pregabalin and dexmedetomidine. |
| **Lubis et al. 2018 [36]** | The tested subjects were randomly allocated into three groups: Group 1: patients administered with a single dose of celecoxib 400 mg and pregabalin 150 mg, Group 2: patients administered with a repetitive dose of celecoxib 200 mg and pregabalin 75 mg twice daily for 3 days before the operation, and Group 3: patients taking a placebo. |
| **Martinez et al. 2014 [37]** | Patients were randomly assigned to receive one of the following four treatments: (i) placebo ketamine and placebo pregabalin (placebo); (ii) intravenous ketamine with a 0.5 mg.kg_1 bolus at the time of anaesthesia induction immediately followed by a 3 lg.kg_1.h_1 infusion stopped at skin closure and placebo pregabalin (ketamine alone); (iii) oral pre-operative pregabalin 150 mg and placebo ketamine (pregabalin alone); (iv) the combination of these same doses of ketamine and pregabalin (ketamine and pregabalin) at the moment of anesthesia. |
| **Mathiesen et al. 2008 [38]** | Three groups: one group received placebo; second group: pregabalin 300 mg orally; and third group: pregabalin and dexamethasone (8 mg), before the induction of anaesthesia. One hour before anaesthesia and according to their groups, patients received pregabalin 300 mg or placebo orally. |
| **Niruthisard et al. 2013 [39]** | In the morning of surgery, patients randomly received placebo (lactose), pregabalin (150 mg) and placebo, celecoxib (400 mg) and placebo, or pregabalin (150 mg) and celecoxib (400 mg) 1 hour before starting anesthesia. |
| **Singla et al. 2014 [40]** | Patients received pregabalin 150 mg/d (75 mg bid), or 300 mg/d (150 mg bid), or placebo (bid). two preoperative treatment doses at 12 hours and 2 hours before surgery and continued treatment (bid dosing) for 6 weeks post-TKA. |
| **YaDeau et al. 2015 [12]** | Patients received one capsule twice a day until POD14 (total daily dose of 0, 100, 200, or 300mg pregabalin), then one capsule at bedtime on POD15 and POD16. Two capsules were given ∼30 min before transfer to the operating room. |
| **Yik et al. 2019 [41]** | Patients randomised to the intervention group received pregabalin 75 mg orally about 1 h prior to surgery, followed by another 75 mg dose per night for 48 h postoperatively. Patients randomized to the control group received a placebo in place of pregabalin. |
| **Zhou et al. 2023 [42]** | Patients were allocated to one of four groups: placebo group: 200 mg + 150 mg placebo; pregabalin group: 150 mg pregabalin + 200 mg placebo; celecoxib group: 200 mg celecoxib + 150 mg placebo; and pregabalin combined with celecoxib group: 150 mg pregabalin + 200 mg celecoxib. Each group was given the medication program 12 hours before surgery and 2 hours before surgery. |
