# Supplementary figures and images for: Efficacy and safety of pregabalin for postoperative pain after total hip and knee arthroplasty: a systematic review and meta-analysis
Source: J Orthop Surg Res. 2025 Mar 11;20:261. doi: 10.1186/s13018-025-05675-6 (PMC11895303; doi:10.1186/s13018-025-05675-6)

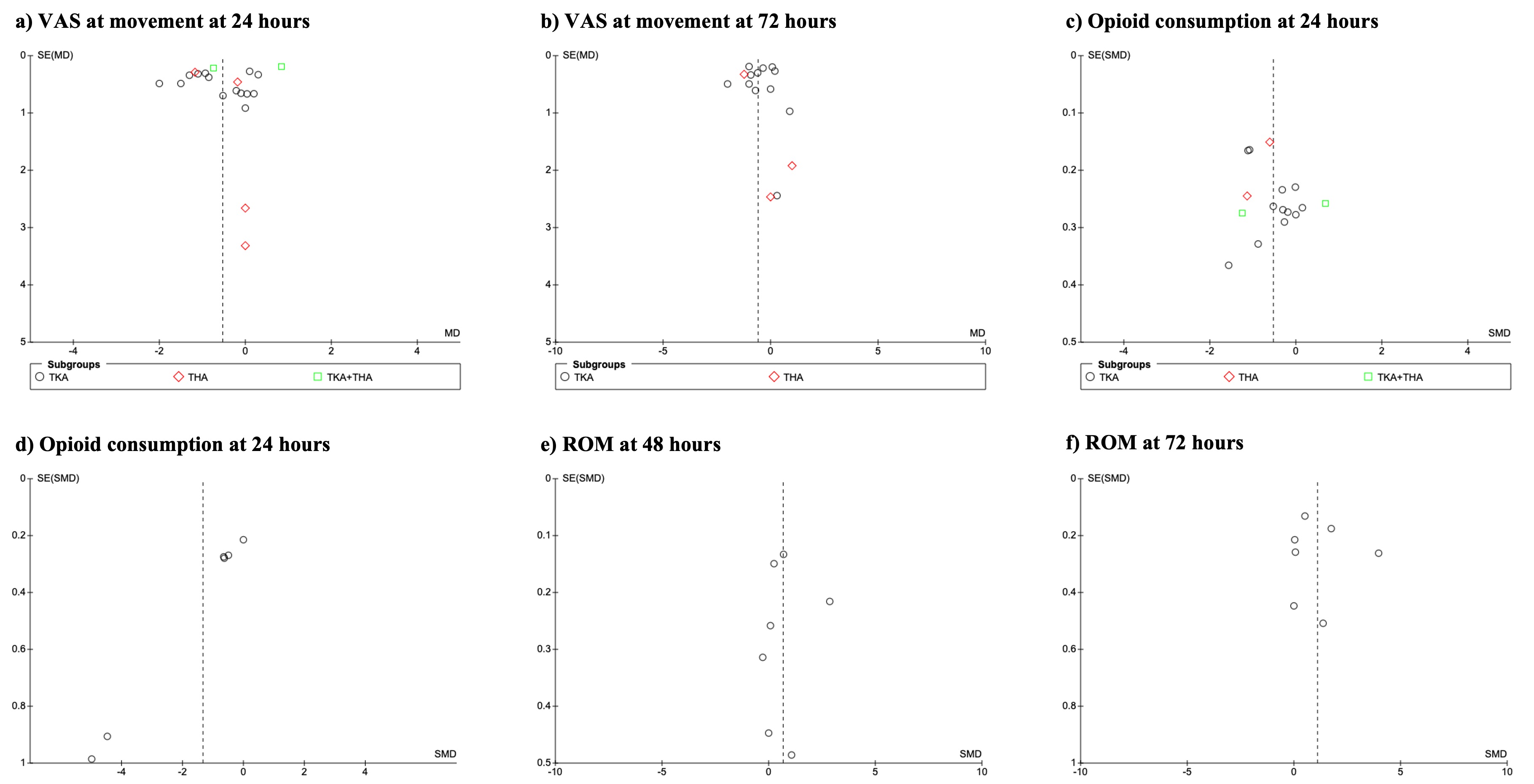

Supplement: Supplementary file 4 — Supplementary Material 4: Additional Fig. 1 file: JPG file showing asymmetry of the funnel plot [file 13018_2025_5675_MOESM4_ESM.jpg]

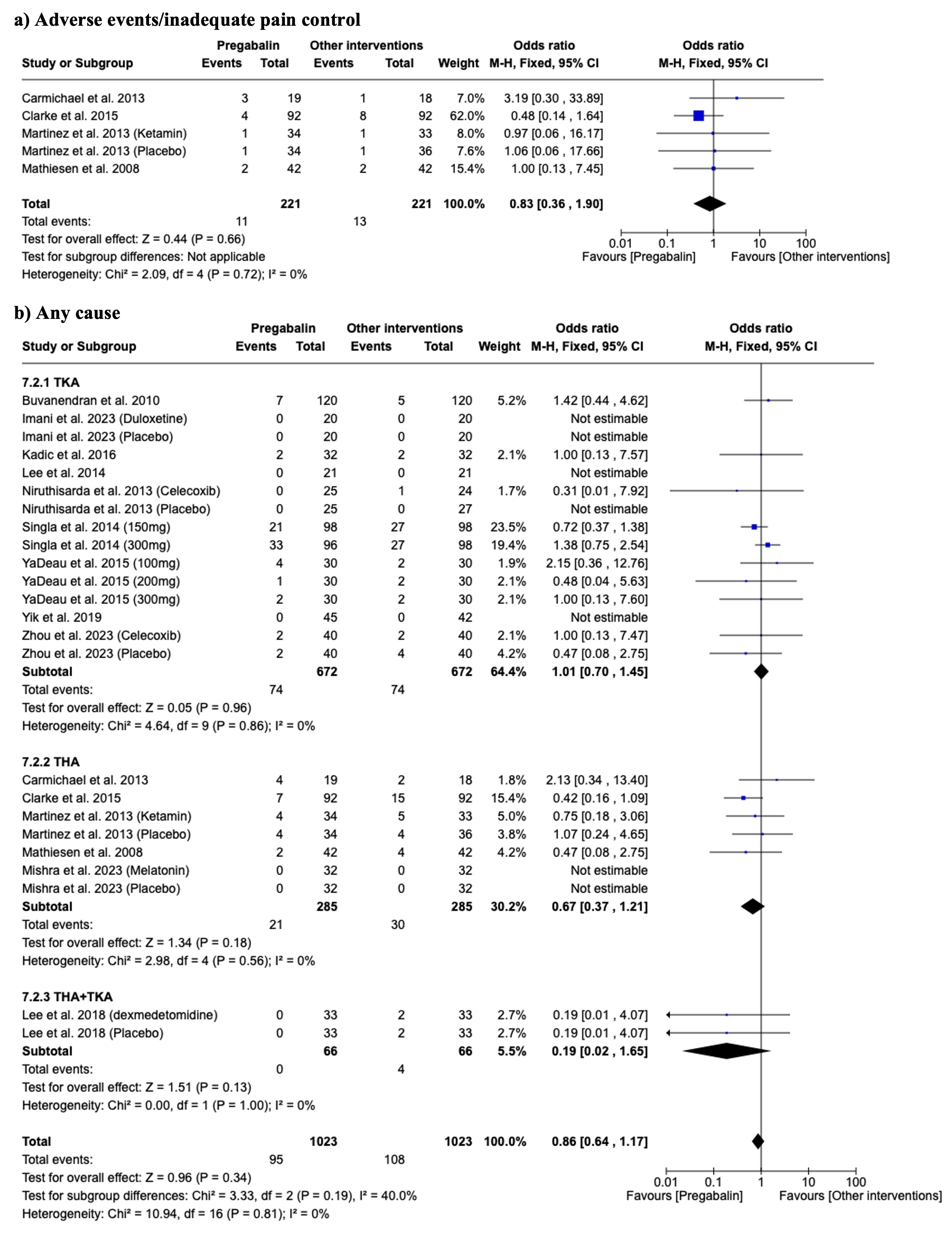

Supplement: Supplementary file 5 — Supplementary Material 5: Additional Fig. 2 file: JPG file of adverse events [file 13018_2025_5675_MOESM5_ESM.jpg]
